# Longitudinal Associations Between Neighborhood Social Cohesion and Sleep Disturbance Among Older Adults: The Mediating Role of Depressive Symptoms

**DOI:** 10.21203/rs.3.rs-11465830/v1

**Published:** 2026-10-23

**Authors:** Qinghong Zhou, Nan Lu, Wanyao Li, Wenrui Yao

## Abstract

Sleep disturbance is highly prevalent in later life and often co-occurs with depressive symptoms. However, the social and psychological factors that contribute to its development remain poorly understood. This longitudinal study examined whether perceived neighborhood social cohesion was associated with subsequent sleep disturbance and whether depressive symptoms mediated this association among older adults. Data were drawn from five waves (2006, 2010, 2014, 2018, and 2022) of the psychosocial subsample of the Health and Retirement Study. The analytic sample comprised 3,360 respondents aged 50 years or older. Univariate and parallel process latent growth curve models were used to examine the developmental trajectory patterns and their associations. A latent growth curve mediation model was further utilized to test the indirect pathways at both the intercept and slope levels. At the intercept level, higher neighborhood social cohesion in 2006 was associated with lower levels of depressive symptoms and sleep disturbance in 2010. The indirect association via depressive symptoms was significant (B = − 0.311, 95% CI [− 0.383, − 0.239]). At the slope level, greater declines in cohesion were associated with greater increases in depressive symptoms (B = − 0.351, 95% CI [− 0.676, − 0.026]), and the indirect path on change in sleep disturbance was also significant (B = − 0.332, 95% CI [− 0.651, − 0.014]). Depressive symptoms may represent an important pathway linking neighborhood social cohesion to sleep disturbance among older adults, highlighting the potential value of strengthening social cohesion and addressing depressive symptoms to promote sleep health.

